# Translating Implementation Experiences and Lessons Learned From Polio Eradication Into a Global Health Course: Insights From an International Consortium

**DOI:** 10.9745/GHSP-D-20-00460

**Published:** 2021-09-30

**Authors:** Anna Kalbarczyk, Svea Closser, Aditi Rao, Oluwaseun Akinyemi, Humarya Binte Anwar, Eric Mafuta, Piyusha Majumdar, Olakunle O. Alonge

**Affiliations:** a Johns Hopkins Bloomberg School of Public Health, Baltimore, MD, USA.; b Department of Health Policy and Management, College of Medicine University of Ibadan, Ibadan, Nigeria.; c BRAC James P. Grant School of Public Health, BRAC University, Dhaka, Bangladesh.; d University of Kinshasa School of Public Health, Kinshasa, The Democratic Republic of the Congo.; e Indian Institute of Health Management Research, Jaipur, India.

## Abstract

Lessons learned from one global health program can inform responses to challenges faced by other programs. One way to disseminate these lessons is through courses. However, such courses are often delivered by and taught to people based in high-income countries and thus may not present a truly global perspective. The Synthesis and Translation of Research and Innovations from Polio Eradication (STRIPE) is a consortium of 8 institutions in Afghanistan, Bangladesh, the Democratic Republic of the Congo, Ethiopia, India, Indonesia, Nigeria, and the United States that seeks to carry out such a transfer of the lessons learned in polio eradication. This short report describes the collaborative process of developing content and curriculum for an international course, the learnings that emerged, the barriers we faced, and recommendations for future similar efforts. Various parts of our course were developed by teams of researchers from countries across South Asia and sub-Saharan Africa. We held a series of regional in-person team meetings hosted in different countries to improve rapport and provide a chance to work together in person. The course content reflects the diversity of team members’ knowledge in a variety of contexts. Challenges to this effort included team coordination (e.g., scheduling across time zones); hierarchies across and between countries; and the coronavirus disease (COVID-19) pandemic. We recommend planning for these hierarchies ahead of time and ensuring significant in-person meeting time to make the most of international collaboration.

## Abstract (precis)

Using international collaborations to develop educational materials presents several challenges but offers enormous benefits in gleaning a wealth of information, perspectives, and context. The global course that resulted from this collaboration mirrors the goals of implementation science more broadly—to bring the findings of research into routine practice to improve health services.

## Teaser

**Key Findings:**

- Using international collaborations to develop educational materials is an effective approach to obtaining a wealth of information, perspectives, and context and to facilitating local and global ownership and uptake of the educational materials.
- While these collaborations can involve challenges for curriculum design, content development, and team cohesion, in-person meetings and early recognition of institutional hierarchies can mitigate challenges and enhance team strengths.

**Key Implications:**

- Collaborations should plan for and adjust approaches for hierarchies ahead of time and ensure significant in-person meeting time to make the most of international collaboration.

## INTRODUCTION

The knowledge amassed in the process of implementing a global health initiative can enhance frameworks for prevention, care, and treatment for a broader range of health conditions. Lessons learned from one program can profoundly shape the response to the challenges faced in other programs. For example, lessons learned from the HIV/AIDS epidemic have been leveraged to address noncommunicable diseases^1^ and Ebola-related stigma.^2^ However, widespread uptake of key public health lessons (both positive and negative) does not occur passively; the knowledge gained from one initiative will not automatically transfer to another without active strategies.^3^^–^^5^ Public health practitioners tend to produce narratives that validate their work, but more complex accounts that integrate an understanding of politics and history are key to truly replicating solutions to global health problems.^6^

A major way of delivering this knowledge is through education, using courses that explore the complexities of lessons learned in ways that allow knowledge gained in one program to benefit others. Courses with a global perspective, however, are often taught by and for residents of high-income countries, leaving out those in low- and middle-income countries (LMICs) who are most in a position to act on lessons learned.^7^ Massive open online courses (MOOCs) are a powerful way to reach a global audience, but most MOOCs on major platforms are developed by academics based in high-income countries, a reflection of the resources required to launch them.^8^ If a MOOC is truly to reach a global audience, we felt that the lessons and experiences that go into its creation and the expertise around teaching reflected in its instructors should be similarly global. Further, many students across the globe would be best reached not by MOOCs, but by in-person courses taught by local experts. Hence, our course was designed by a global team that could deliver both MOOCs and in-person content to reach students in a variety of public health contexts across the world.

The Synthesis and Translation of Research and Innovations from Polio Eradication (STRIPE) project is a collaboration of researchers from 8 countries (Afghanistan, Bangladesh, the Democratic Republic of the Congo [DRC], Ethiopia, India, Indonesia, Nigeria, and the United States) working to collect, synthesize, and disseminate lessons learned from polio eradication activities (Table 1), with advice from a technical advisory committee (TAC) composed of various global stakeholders.^9^ The scale of polio eradication, a global initiative that has been carried out in more than 200 countries and currently has a budget of more than $1 billion a year, has resulted in a wealth of experiences in varied contexts that can be adapted by other health initiatives. Polio lessons are especially relevant for mass vaccination programs aiming to eliminate or eradicate a disease, and there are many direct lessons for coronavirus disease (COVID-19) control efforts as well as measles elimination efforts. But many topics are more broadly relevant: global agenda setting, data collection and management, social mobilization, and human resource management, to name a few.

**TABLE 1.** Synthesis and Translation of Research and Innovations From Polio Eradication Project Consortium Partner Institutions

**Partner Institution and Country**
1. Global Innovations Consultancy Services, Afghanistan 2. BRAC James P. Grant School of Public Health, BRAC University, Bangladesh 3. School of Public Health, University of Kinshasa, the Democratic Republic of the Congo 4. School of Public Health, Addis Ababa University, Ethiopia 5. Indian Institute of Health Management Research University, India 6. Faculty of Medicine, Public Health and Nursing, Gadjah Mada University, Indonesia 7. College of Medicine, University of Ibadan, Nigeria 8. Johns Hopkins Bloomberg School of Public Health, USA

We aim to bring the lessons and experiences from the polio eradication effort into academic and training programs for various global health audiences and thus facilitate the active transfer of tacit knowledge from polio to global health actors, students, and other health initiatives. We used mixed methods to collect lessons learned from diverse stakeholders who have been involved in the planning and implementation of polio eradication activities at the global level and national and subnational levels in the 7 partner countries.^9^ We are disseminating our findings through a variety of channels. This article focuses on our global health course, which explores lessons learned from polio eradication through the lens of implementation science. The goals of the course mirror the goals of implementation science more broadly—to bring the findings of research into routine practice to improve health services.

This article focuses on our global health course, which explores lessons learned from polio eradication through the lens of implementation science and ultimately to bring the findings of research into routine practice to improve health services.

The course is available online, free of charge, and as several MOOCs hosted on the project website and FutureLearn (a digital education platform), enabling global online access. The course is also being delivered in person by the STRIPE consortium members across the world. The course materials, including lecture recordings and PowerPoint slides, are freely available for use by instructors who wish to integrate this material into existing courses. This short report describes the collaborative process of developing the course with academic partners from institutions across both LMICs and high-income countries, and it outlines our lessons learned from such collaborative processes.

## THE PROCESS OF CREATING AN ADAPTABLE COURSE WITH GLOBAL REACH

The experiences of polio eradication have relevance across a wide range of global health theory and practice, from global alliance building to surveillance systems to community engagement to health equity. We determined the topics to include in our course during a 2-day consortium-wide meeting held in April 2019, in Baltimore, Maryland, USA, where researchers from all consortium institutions came together to present and discuss initial findings from our research. Before the meeting, we listed potential topics to be covered in the course, drawing both on the global health literature^10^ and preliminary data emerging from our project. We then completed a consensus-building exercise during the meeting to determine which of these topics we should include. The consortium agreed on a final set of 10 knowledge domains (Table 2).

**TABLE 2.** Knowledge Domains Included in Global Health Course on Polio Eradication

**Global Health Knowledge Domains**	**Domain Overview** ^a^
1. Global alliances	History of the global polio eradication alliance and its trajectory. Provides strategies for alliance building, focusing on how approaches differ depending on health systems, structures, and political contexts.
2. Policy engagement and influence	Defines policy engagement and its importance in global disease control programs. Explores the importance of process, stakeholders, and contexts in considering policy engagement approaches.
3. Field epidemiology and outbreak response	Explores surveillance as a decision support system for planned as well as outbreak responses.
4. Data for decision making	Explores various data collected through polio eradication activities and how they are interpreted to support program decision making. Considers factors that influence the quality of data.
5. Health communications and behavior change	The role of health communications for behavior change and the key principles in designing communication tools, focusing on how they are adapted and refined across different phases of a program and across contexts.
6. Community engagement	Importance of community engagement in health programs and strategies for engaging communities. Considers its key role in long-term health programming and trust building.
7. Planning and management	Considers key elements of planning and management, including microplanning and monitoring.
8. Health commodities, logistics, and supply	Importance of and best practices in logistics management for public health programs, especially large-scale and multicountry programs.
9. Human resources for health	Considers who polio workers are and the challenges they face in achieving disease control, as well as critical factors to consider in acquiring, training, and deploying appropriate health care workers.
10. Health equity and social justice	Describes social determinants of health and how global disease control programs shape global equity and equality. Considers the impact on hard-to-reach populations.

a Materials for all domains can be found here: https://stripe.jhu.edu/learning-hub/global-health-course/.

We felt strongly that this course should be driven by experienced researchers and educators based in the domains described in the course who were familiar with the diverse educational contexts in which the course would be taught. We also decided that, given the interdisciplinary nature of implementation science and relevance for diverse global health programs, each knowledge domain in the course should teach relevant core competencies in implementation science.^11^ This decision led to a process of international collaboration across contexts, which facilitated diverse participation but was logistically challenging. The Figure presents the overall timeline of this process.

**FIGURE:**
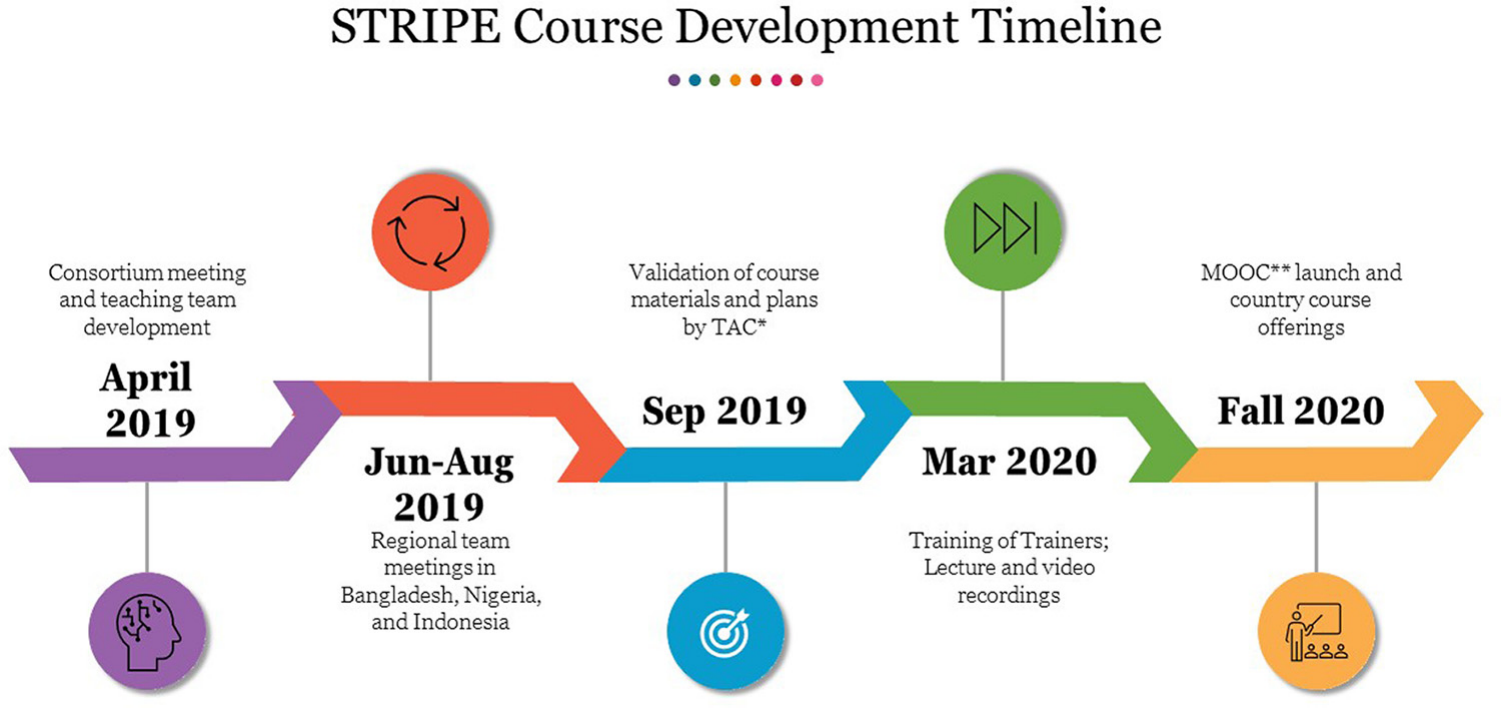
Synthesis and Translation of Research and Innovations From Polio Eradication (STRIPE) Course Development Timeline MOOC, massive open online course; TAC, technical advisory committee.

The process of international collaboration across contexts facilitated diverse participation but was logistically challenging.

### Team Development

At our initial meeting in April 2019, we distributed a brief survey asking consortium researchers to identify topic areas from the course in which they had expertise, interest in developing curriculum, and substantial familiarity with the data from our research project. We used the results of the survey to form “teaching teams” for each topical area, which were then tasked with working together to develop the curriculum for that topic. We sought to achieve gender and geographical representation (i.e., members from both Asian and African countries) in each team. This was the first point in the overall project when individuals from different countries were asked to work extensively together and not just with others within their country teams. Each teaching team was assigned a facilitator from Johns Hopkins University (JHU) who had knowledge of that learning domain and expertise in curriculum development.

### Regional Meetings

We held 3 regional meetings to facilitate curriculum development. The first meeting was held in Dhaka, Bangladesh, in June 2019, following the Global Conference on Implementation Science and Scale-up. Members from each of the consortium institutions were invited to attend; in addition to JHU members, 4 of the 7 partner institutions were represented (from Bangladesh, India, Indonesia, and Nigeria). During this meeting, we discussed course design, expected outputs, and proposed timelines for completing drafts of lectures and case studies. Teaching teams met to discuss their knowledge domains, develop outlines for the lectures, brainstorm potential case studies based on their data, and divide tasks among team members. We shared this information with partners who could not be present for feedback.

The second regional meeting was held in early August 2019 in Ibadan, Nigeria. Before the meeting in Nigeria, teaching team members submitted draft lectures. In addition to providing feedback on these lectures, attendees also reviewed 1 case study, which prompted discussion on how to change existing draft cases. The teams set the next set of deadlines in preparation for the final regional meeting.

The third regional meeting was hosted in Yogyakarta, Indonesia, in late August 2019. At this meeting, we discussed our varied experiences with the case-based learning method. Small groups met to review drafts of lectures, outline major revisions, and identify gaps where additional research data needed to be incorporated. This meeting was attended by collaborators from Afghanistan, Bangladesh, India, Indonesia, and the United States.

### Validation

Each teaching team finalized their lecture materials, accompanying teaching notes, and case studies in preparation for a TAC meeting in September 2019. The TAC is composed of individuals representing faculty from schools of public health, Ministries of Health, and nongovernmental organizations in low-, middle-, and high-income countries, as well as experts representing core Global Polio Eradication Initiative partners (World Health Organization, United Nations Children’s Fund, Bill & Melinda Gates Foundation, U.S. Centers for Disease Control and Prevention, Rotary International). At the meeting, TAC members provided useful feedback on key lessons learned to include and highlight in the course (e.g., to highlight specific experiences relevant for the lecture content, redescribing content to appeal to a diverse global audience). TAC members subsequently reviewed the course material relevant to each of their areas of expertise.

### Training of Trainers

A final meeting was held in Baltimore in March 2020, attended by collaborators from Afghanistan, Bangladesh, DRC, Ethiopia, Nigeria, and the United States, while members from India joined remotely. During the meeting, we discussed and finalized course structure and flow, identified course assignments and activities, and shared strategies on teaching techniques for the on-site course. In addition, lectures and discussions for the MOOC were recorded with support from Johns Hopkins Bloomberg School of Public Health’s Center for Teaching and Learning (CTL). CTL provides training, instructional design services, recording studios, and support for online course development—the sort of MOOC infrastructure found most often at well-resourced institutions.

Subsequently, CTL along with the JHU team formatted and standardized all course materials, including lecture slides, recordings, and case studies, and then packaged the materials both to distribute online and to meet the needs of individual country team’s in-person iterations of the course.

## DESIGNING CURRICULUM AND DEVELOPING CONTENT

One major challenge in developing a course to be taught in so many different settings to many diverse groups of learners is that the background knowledge of those learners will vary widely. To engage and reach a diverse student body, we aimed to create an adaptable course that instructors could tailor both to their region and their students’ experience levels. Hence, for each major topic listed in Table 1, we developed 2 lectures summarizing some major lessons learned from polio in that area. One lecture is appropriate for students with little or no background in the topic in question and uses polio examples to illustrate key basic concepts. For example, the introductory lecture on human resources for health covers basic concepts in the area through the example of the polio workforce. A second lecture in each topic area delves deeper into more complex lessons learned applicable to other health programs. It is designed to be used as a stand-alone lecture for advanced students or as a follow-on to the basic lecture in each topical area. This second lecture has many more opportunities for student reflection, engagement, and application to other health programs. For example, the second lecture on human resources for health explores issues of gender, incentive structure, and lethal violence, all of which have been complex issues for polio eradicators in many contexts. Thinking through these issues has direct utility for the design and implementation of other health programs. The MOOCs include both the basic and the advanced material, so that less experienced learners have the background they need, while more experienced learners can access key information.

One major challenge in developing a course for many different settings and diverse learners is that the background knowledge of the learners will vary widely.

All lectures draw on core learning themes and competencies in implementation science. We paired each lecture with readings to extend knowledge—open-access readings for the MOOCs and a broader range for in-person courses. We also developed case studies for each topic area that dig deeper into implementation challenges in a particular context. They provide students a chance to apply and extend the material presented in lectures, or they can be used alone with more advanced students or in other contexts. Both the lectures and the case studies include a range of perspectives shared with us during the research we did for this project, from global-level policymakers to frontline workers. Table 3 provides links to access each MOOC in addition to a description, the core learning themes, and competencies addressed.

**TABLE 3.** Global Health Polio Eradication Core Course Themes and Implementation Research Competencies for Each Massive Open Online Course^a^

**Modules**	**Course Themes**	**Implementation Research Competencies**
I. Planning and Managing Health Programs: Promoting Quality, Accountability, and Equity		
— Planning and management — Health commodities, logistics, and supply — Human resources for health — Health equity and social justice	— Reaching hard-to-reach populations — Importance of politics — Potential and demands of eradication programs	— Understanding the interventions, their mechanisms, and emerging implementation challenges — Building an effective team — Applying ethical principles in conducting implementation research
II. Building Alliances in Global Health: From Global Institutions to Communities		
— Global alliances — Policy engagement and influence — Health communications and behavior change — Community engagement	— Importance of context — Relationship between vertical programs and health systems — Power of engagement	— Understanding contexts (health systems, implementing organizations, and communities) — Identifying and engaging relevant stakeholders — Leveraging required resources for conducting implementation research — Communicating and advocating effectively throughout the implementation research process.
III. Collecting and Using Data for Disease Control and Global Health Decision Making		
— Field epidemiology and outbreak response — Data for decision making	— Promise and limits of technology — Importance of incentives	— Formulating appropriate implementation research questions — Determining applicable study design and methodology — Conducting research in a robust and rigorous manner — How to use information from implementation research

a The three massive open online courses are available at: https://www.futurelearn.com/partners/stripe.

## INTERNATIONAL ACADEMIC COLLABORATIONS: RICH AND REWARDING

The process of working collaboratively with colleagues across the world facilitated diverse perspectives and learning exchanges within the consortium, which was apparent from the very early days of our collaboration. For example, during the first regional meeting in Bangladesh, small groups worked on developing lecture outlines for each module. As the policy engagement group discussed potential material, 2 members shared how their unique Ministry of Health governance structures either hindered or supported priority setting for polio.

As our familiarity with each other deepened over multiple in-person meetings, these conversations also deepened. By the time we met to record course material for the MOOC, we had thoughtful and insightful conversations about complex topics including corruption; international influence and aid; community opposition to externally funded health programs; and implementing health programs in settings characterized by complex violence. The opportunity to have these conversations across such a breadth and depth of expertise and perspective was a valuable experience for many of us that we were able to transfer to course materials including a series of short video discussions. These video discussions are in the MOOCs and also available separately online for classroom use (Tables 2 and 3).

Beyond topical discussions, learning about and incorporating teaching methods and styles from different institutional contexts constituted an interesting and valuable exercise. For example, at the meeting in Indonesia, we had discussions about the very different ways many of us had previously used case studies in the classroom. These discussions gave all of us, no matter how extensive our previous experience, new tools to use in our teaching going forward.

## INCORPORATING PRACTITIONERS’ PERSPECTIVES

The majority of consortium members currently hold academic positions. Although many of us have worked on polio eradication in various capacities in the past, none of us currently have a full-time job within polio eradication. This carries with it an attendant limitation; although we have all been involved in this current research project on polio eradication, most of us are less familiar with the specifics of day-to-day polio work, even within our own countries, than people who are working full time on polio eradication activities. Thus, we found the experience, review, and contributions of the TAC extraordinarily valuable. TAC members were often able to provide additional insight into the topics we covered in the course, as well as additional resources to share with students.

Our positions as academics do carry an advantage. We are free to discuss difficult and politically sensitive topics in ways that those who work for United Nations agencies and governments would not be able to. Thus, in our course, we were able to openly delve into complex and thorny issues such as the power relations of international aid; the politics of international and local agenda setting; and the ethical trade-offs involved in the goal of eradication. Some of these issues were captured in the video discussions included as part of the MOOC.

## COORDINATING A COLLABORATIVE PROCESS

In addition to being rewarding, our collaboration across countries and continents also bore challenges. Time differences, poor internet connectivity in many areas, and distance made scheduling calls and setting deadlines difficult. As we were on different sides of the world, regular scheduling and follow-up were of foremost importance to maintain a cohesive approach, yet such scheduling and follow-up were challenging. Team members needed to speak to each other regularly to ensure all materials were consistent, built on one another, and met the learning objectives; yet these conversations were often difficult to have online.

Our final meeting happened just as COVID-19 was beginning to spread globally; partners from 6 countries met together in Baltimore, but those of us in India attended via Zoom, an online teleconferencing tool. It was of course not quite the same as being in the same room, especially since the meeting took place in what in India was the middle of the night. Yet we made this collaboration work by the commitment of partners across the world investing their time and energies on advancing the project.

## FORMULATING INTERNATIONAL TEAMS

One critical issue we did not consider when we developed our international teaching teams was that these collaborations were layered on top of existing hierarchies. As described earlier, each teaching team was purposefully created to have geographic and gender representation in addition to expertise, interest, and experience with the topic. However, as teams began to develop materials, it was clear that in some countries, particularly in Asia, our assumption that everyone worked entirely independently was not correct. For example, high-ranking professors occasionally delegated the responsibility for producing lecture materials or case studies to lower-ranked staff in their own countries, even though the latter individual had not attended the teaching team calls. Also, some lower-ranking professors were uncomfortable sharing materials developed with other people on their teaching team until it had been vetted by their in-country superiors. These decisions, while all understandable, made working across countries more challenging. This was especially true where people were working on multiple teaching teams. In the future, we would consider these dynamics more carefully at the outset and plan for them.

Each teaching team was created to have geographic and gender representation in addition to expertise, interest, and experience, but other factors also needed consideration.

Another related issue is that we had many high-ranking and busy professors on our teams and fewer people with substantial time to devote to course development. As a result, sometimes there were many people providing ideas and direction but fewer people to produce content. In practice, this situation meant that we benefited from a wealth of knowledge and valuable feedback but suffered from a shortage of people incentivized to carry out those edits in content production, editing, writing, and so on. This issue would be easy to fix with some advance planning—for example, hiring staff to carry out some of these tasks for teaching teams would have been valuable.

Unique teaching styles, borne out of contextual experience, also complicated the collaborations. Some draft lecture materials were full of text while others utilized imagery. Some members were familiar with the development and use of teaching notes or case-based approaches while others were not. These diverse approaches sometimes made creating cohesive content more difficult. Ultimately, we feel that this diversity of background enriched the course, but again, making materials standardized was labor-intensive.

Because the teams were funded through subcontracts with JHU, yet another hierarchy existed within the consortium. The course development was managed by JHU faculty who made major decisions about course content and direction. This circumstance added another layer of hierarchy and was unfortunately a different dynamic than if the project had been managed and run out of one of the LMIC institutions in our consortium. One benefit of centering the project at JHU was access to resources. For example, we had access to CTL, a global leader in instructional design for different audiences that has a wealth of experience in creating aesthetic and accessible online content. But this particular hierarchy mirrored larger power differentials globally and at times created tension. Having the administration of the course centered in one of the LMIC institutions rather than at JHU would have potentially alleviated some of these issues.

## RECOMMENDATIONS

Academic collaborations across countries are well worth the challenges outlined, but anticipating and considering how to mitigate some of these issues ahead of time would be helpful. Of note, hierarchies both between senior and junior faculty and between primary and sub awardees should be considered and planned for at the outset. Wherever possible, in-person international meetings should be held to facilitate collaboration, motivate team members, and facilitate relationship development/maintenance. Investing in developing team rapport helps to foster a collective understanding, allows for nuanced exchange of ideas and approaches, and results in greater cohesion and depth of thought in the output. We also recommend providing substantial staffing support to execute the insightful ideas of senior faculty. This infusion of human resources is needed throughout the process, from conceptualization to curriculum design and to content development and delivery.

## CONCLUSIONS

Using international collaborations to develop educational materials is an excellent approach to obtaining a wealth of information, perspectives, and context. This approach is particularly important in implementation science where context is king. While exceptionally valuable, these collaborations can be challenging to organize, and in-person meetings are needed to keep materials cohesive. Hierarchical contexts, both within each country and across consortium members, should be considered in the planning phases and this may mean adjusting expectations and workload balances.

Overall, though, the benefits of collaborating to build a course across countries and contexts were enormous. All of us learned an extensive amount from our colleagues about health program implementation in contexts ranging from the remotest corners of DRC to the center of Jakarta, Indonesia. Not only did this lead to rich course material, but it also led to the formation of new collaborations across continents that many of us hope to build on throughout our careers.

## Notes

**Peer Reviewed**

**First published online:** July 19, 2021.
